# Network Pharmacology Approach to Uncover the Mechanism Governing the Effect of Radix Achyranthis Bidentatae on Osteoarthritis

**DOI:** 10.1186/s12906-020-02909-4

**Published:** 2020-04-21

**Authors:** Laigen Zhang, Xiaoqing Shi, Zhengquan Huang, Jun Mao, Wei Mei, Liang Ding, Li Zhang, Runlin Xing, Peimin Wang

**Affiliations:** Department of Orthopaedics and Traumatology, The First Affiliated Hospital of Nanjing University of Chinese Medicine, Jiangsu Provincial Hospital of Traditional Chinese Medicine, No 155, Hanzhong Road, Nanjing, 210029 Jiangsu Province China

**Keywords:** Network pharmacology, Mechanism prediction, Radix Achyranthis Bidentatae

## Abstract

**Background:**

This study used a network pharmacology approach to elucidate the molecular mechanism governing the effect of Radix Achyranthis Bidentatae (RAB) on osteoarthritis (OA).

**Methods:**

Based on oral bioavailability and drug-likeness, the main active components of RAB were screened via the Traditional Chinese Medicine Systems Pharmacology platform. The GeneCard, OMIM, PharmGkb, Therapeutic Targets database, and DrugBank database were used to establish a database of osteoarthritis targets. The interactive active network map of “ingredient-target” was constructed with Cytoscape software (Version 3.7.1). The protein-protein interaction network was constructed with the STRING database, and the related protein interaction relationship was analysed. GO biological function analysis and KEGG enrichment analysis for core targets were performed. Finally, docking of the active components with the core target was carried out.

**Results:**

Sixteen active components of RAB were obtained, and 63 potential targets for OA were identified. Network analysis results indicate that these targets are primarily involved in regulating biological processes, such as cell metabolism, apoptosis, and cell proliferation. Pathways involved in the treatment of osteoarthritis include virus-related signalling pathways, apoptosis signalling pathways, IL-17 signalling pathways, and PI3K/AKT signalling pathways.

**Conclusion:**

RAB has the characteristics of being multi-system, multi-component and multi-target. Possible mechanisms of action for RAB include regulating the immune and inflammatory responses, reducing chondrocyte apoptosis, and protecting the joint synovial membrane and cartilage to control disease development. The active ingredients in RAB, such as sterols and flavonoids, exhibit strong potential as candidate drugs for the treatment of osteoarthritis.

## Background

Osteoarthritis (OA) is a common chronic disease with a significant impact on human health. With the ageing of the general population, the incidence of OA has been increasing annually [1]. In 2014, 13% of the elderly were diagnosed with OA according to population-based healthcare data from England and Sweden [2]. In medical findings concerning people over 55, approximately 25% of the population reports at least one knee pain attack every year, which is likely to reflect the potential of OA [3]. Pain and dysfunction significantly reduce patients’ quality of life and cause serious social and economic burdens. In the statistics regarding years living with disability, OA ranks first among non-infectious diseases [4].

Chinese herbal medicine (CHM) has a long history and unique features for the treatment of OA; CHM is observed to be effective in the improvement of clinical symptoms, such as pain and stiffness, with few side effects and lower costs than Western medicine [5–8]. Pan and Huang [9, 10] found that Radix Achyranthis Bidentatae (RAB) is employed most frequently in the treatment of knee osteoarthritis ranked first among other herbs.

The source of *Achyranthes bidentata* is the root and rhizome of *Achyranthes aspera* L., which is a traditional Chinese medicinal plant in China [11]. *Achyranthes bidentata* is widely distributed in tropical and subtropical regions. According to the ancient books of traditional Chinese medicine, the main effects of RAB include nourishing the liver and kidney, strengthening bones and muscles, and invigorating circulation. Modern medicine’s research on *Achyranthes bidentata* primarily focuses on the biological effects on the immune system, nervous system, and bone metabolism, as well as antitumour, antioxidation, and joint-protection properties [12, 13]. Polysaccharides, polypeptides, alkaloids, triterpenoid saponins, organic acids, ketosteroids and various trace elements are known as the primary classes of bioactive ingredients [12].

*Achyranthes bidentata* Bl. saponins (ABS) can not only promote osteogenic differentiation of bone marrow mesenchymal stem cells by activating the ERK signalling pathway but also protect chondrocytes by inhibiting the activation of NF-κB in rat cartilage induced by IL-1B, thereby inhibiting IL-1B-induced apoptosis [14, 15]. *Achyranthes bidentata* polysaccharides (ABPs) promote the proliferation of chondrocytes and inhibit the production of osteoclasts, possibly by activating the Wnt/β-catenin signalling pathway and inhibiting RANKL signalling [16, 17]. In addition, other studies have shown that *Achyranthes bidentata* alcohol promotes the proliferation of osteoblasts in a dose-dependent manner, possibly by activating the ERK signal transduction pathway to stimulate osteoblast differentiation [18].

The pharmacological study of ARB and the mechanism of its treatment of OA have been investigated by a number of studies. However, the drug compositions of ARB are complex, and the specific mechanism of its treatment of OA still needs to be fully elucidated. CHM treatment of diseases has the characteristics of “many-to-many-to-many”, that is, the complex process of multiple targets to multiple channels to multiple genes. At present, it remains difficult to explore the potential molecular mechanism of CHM because of the diversity of its components and the complexity of its interactions with the human body. Traditional single-agent or monomer studies were unable to fully explain the specific mechanism of CHM treatment of certain diseases. Based on the development of systems biology and bioinformatics, modern technology has entered the era of omics and big data. Researchers have attempted to explore the correlation between diseases and drugs from the perspective of network biology. In 2007, Chinese scholar Li Shao et al reported the network regulation effects of the TCM syndrome biomolecular network and proposed a biomolecular network to study the active compounds of Chinese medicine [19]. Then, based on the theory of systems biology, Hopkins first proposed the term “network pharmacology.” Hopkins explained the relationship between drugs and the body from the perspective of improving or restoring the balance of biological networks and built a “drug-targeted disease” network [19].

Network pharmacology is based on the “disease-gene-target-drug” interaction network, which systematically and comprehensively characterizes the intervention and influence of drugs on the disease network, thereby elucidating the synergistic effects of drugs on the human body. The booming development of bioinformatics provides the possibility to clarify the mechanism of TCM for the treatment of diseases.

**Fig. 1:**
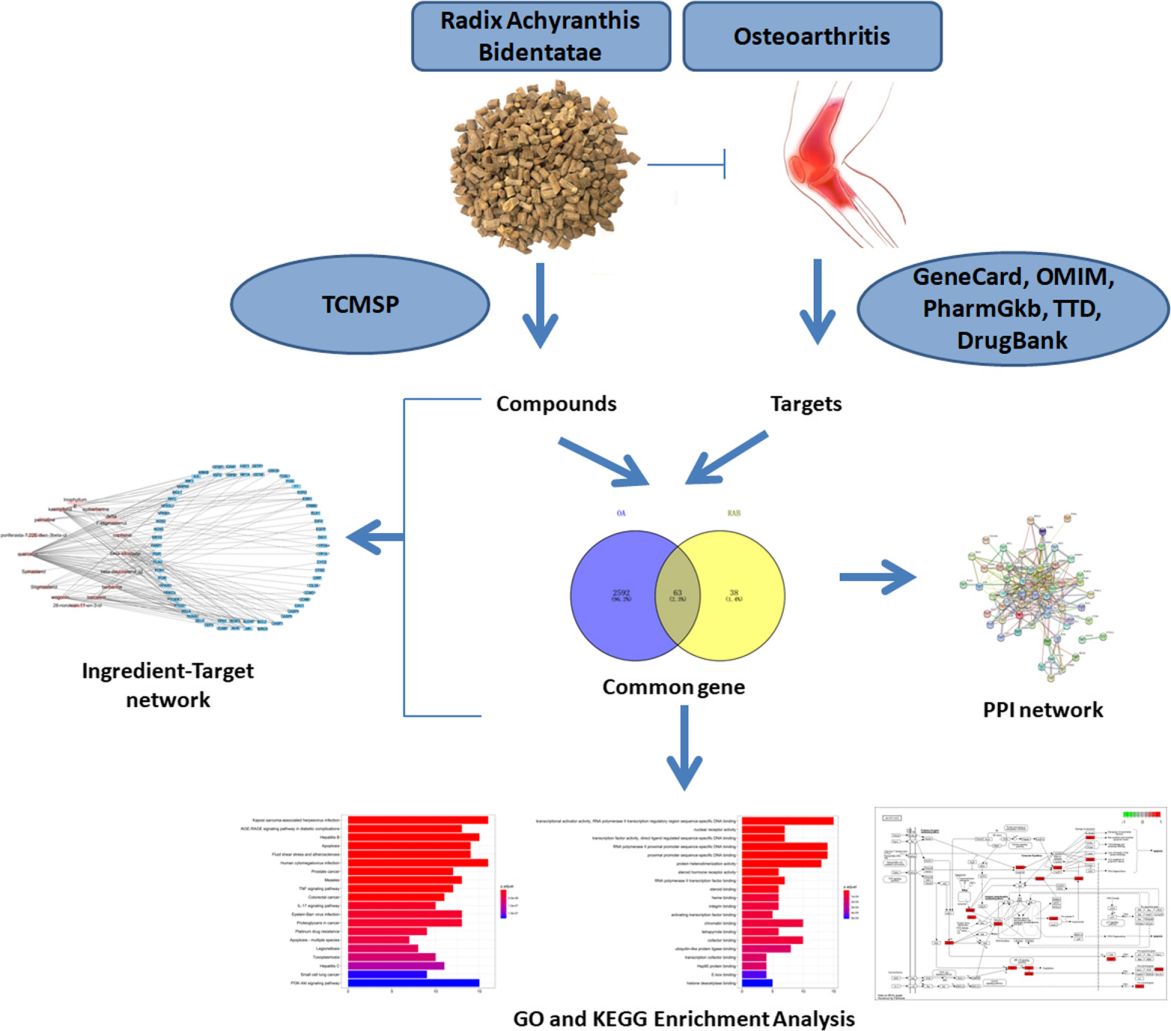
Whole framework based on network pharmacology

Based on the network pharmacology approach, this study focuses on the mechanism governing the effect of RAB on OA at the molecular level, attempts to elucidate the specific targets and molecular signalling pathways of RAB acting on OA, and devises new strategies for drug development and clinical applications. A flowchart of this study is depicted in Fig. 1.

## Methods

### Compound collection and screening

Traditional Chinese Medicine Systems Pharmacology (TCMSP) is a unique system pharmacology platform of CHM that captures the relationships among drugs, targets and diseases [20]. In TCMSP, we retrieved the chemical composition information of RAB. The active ingredients of RAB were obtained from the screening conditions as follows: oral bioavailability (OB) > 30% and drug-likelihood (DL) > 0.18 [21, 22]. Then, the potential targets of RAB were collected through the TCMSP platform. With the UniProt database (https://www.uniprot.org/uniprot/), the RAB gene target was obtained.

### Target acquisition for drug and disease-related effects

Using osteoarthritis as a keyword, the OA gene was searched and screened by the following electronic databases: GeneCard database (https://www.genecards.org/), OMIM database (https://www.omim.org/), PharmGkb database (https://www.pharmgkb.org/), Therapeutic Targets database (http://bidd.nus.edu.sg/BIDD-Databases/TTD/TTD.asp), and DrugBank database (https://www.drugbank.ca). The database search results are combined, and duplicate targets are deleted to obtain all the target targets of OA (the results are shown in Supplementary Table 1). RAB matched the target prediction results of active ingredients with the retrieval results of OA-related targets and selected overlapping targets as the related targets of RAB for the treatment of OA. The Venny 2.1 (http://bioinfogp.cnb.csic.es/tools/venny/index.html) online tool was used to map the active ingredient target and the disease target of RAB and to draw Venn diagrams. Then, the intersection targets of RAB and OA were collected as the common genes of RAB in the treatment of OA, which might be the potential target set of RAB in the treatment of OA.

### Construction of drug active ingredients and disease target network

The screening results of the active components of RAB and OA targets were imported into Cytoscape [23], and the active components and OA target network of RAB were constructed. The Network Analyzer Apps software was then used to analyse the topology properties of the network, including degree, betweenness centrality, and closeness centrality.

In this experimental study, degree refers to the number of direct neighbours of a node. It is considered that the greater the number of directly connected nodes of a node are, the greater the influence is [24]. Betweenness centrality measures the shortest path between all pairs of nodes through the node [25, 26]. Closeness centrality is defined as the reciprocal of the average of the shortest path distance between a node and all other nodes in the network, and the larger the value is, the greater the centrality of the node is, indicating that the signal is passed from one node to other nodes faster.

The nodes of the network represented the target or active component, and the edges indicated their interaction. The core architecture of Cytoscape software was the network. Each node was a protein or an active component. The edge between nodes represents the interaction between these biomolecules. The degree of nodes represents the number of nodes connected with one another in the network. A node with a high degree of betweenness centrality and closeness centrality values means that the node plays a highly important role in the network.

### Protein-protein interaction (PPI) network construction and core gene screening

PPI refers to the process in which two or more protein molecules form protein complexes through noncovalent bonds. PPIs and acquired networks are highly important in most biological functions and processes [27]. The STRING database (https://string-db.org/) collected a large number of protein interactions and contains a range of data confidence (low confidence: < 0.4; medium confidence: 0.4–0.7; high confidence: > 0.7). On this basis, common gene targets of RAB and OA were imported into the STRING database. Using the species-qualified *Homo sapiens*, a confidence level of 0.7 and hidden disconnected nodes in the network, a protein interaction network analysis was carried out, and the TSV format of the updated results was downloaded. The file was imported into Cytoscape for topology attribute analysis. We then assessed the topological property of nodes in the interaction network by calculating six parameters with the App CytoNCA [28]: “degree centrality (DC),” “betweenness centrality (BC),” “closeness centrality (CC),” “eigenvector centrality (EC),” “network centrality (NC),” and “local average connectivity (LAC).” These six parameters measured the importance of nodes in the network and indicate the nature of the nodes in the network. A node with high DC, BC, CC, EC, NC and LAC values means that it plays a highly important role in the network. Based on the results of topological property analysis of the above PPI, targets above the median were selected as the core targets and screened twice [21]. Based on the above findings, we obtained the core target in the network.

### GO and KEGG enrichment analysis

A variety of bioinformatic analyses and visualization of the results can be achieved using the software R project. First, the Entrez gene ID of the core target was obtained by RGUI and org. Hs.eg.db, and then the GO function enrichment (Molecular Function, MF, Biological Process, BP, Cellular Component, CC) and KEGG pathway were enriched by RGUI, DOSE and Cluster Profiler. In the programming language, pvalueCutoff = 0.05 and qvalueCutoff = 0.05 were set. The results of the analysis selected the top 20 items with the highest enrichment and displayed them in the form of bar graphs. Finally, using RGUI and pathview, the top 20 access maps of the enrichment rankings were downloaded through the KEGG database (KEGG, https://www.kegg.jp/kegg/pathway.html, updated on July 1, 2019). The results were screened repeatedly and manually selected based on relevance.

### Acquisition of drug-like components and OA target crystals

Through the construction of the target network of the active ingredient of the drug and the disease, the drug-like component of the RAB and the target of OA were obtained. The mol2 structure of the abovementioned drug-like component, celecoxib (positive control drug), was downloaded from the Chemical Book database (https://www.chemicalbook.com/). The protein crystal structure of the OA-related target was downloaded from the RCSB PDB database (https://www.rcsb.org/).

### Docking steps and results evaluation

The results obtained in 2.6.1 were sequentially imported into Discovery Studio 2016 3.0 software for pre-processing. First, Prepare Ligands is applied to each drug component, and the parameters are default values and saved as molecular docking ligands. Second, through Prepare Protein, the parameters take the default value, the pre-treated protein structure is obtained, the original ligand is extracted from the target, and the molecule is paired as an acceptor. The receptor active site selects the original ligand extraction position, the docking preference is set to high quality in the LibDock module, the conformation method is BEST, parallel processing is true, and other parameters take the default values. The docking results are given by the scoring function LibDock score. The higher the LibDock score is, the higher the activity of the predicted component binding to the target is.

## Results

### Screening of active ingredients in RAB

The TCMSP database was searched using the screening conditions OB (> 30%) and DL (> 0.18). A total of 20 active ingredients were found in RAB. Twenty active ingredients were manually searched for related targets, and 16 active ingredients were finally selected, as detailed in Supplementary Table 2. Among these ingredients, phytosterols, such as stigmasterols, berberine and other alkaloids, have been proven to have anti-inflammatory, antioxidation, anticancer and other broad pharmacological effects [29–32]. Flavonoids, such as quercetin and baicalein, can eliminate cartilage degeneration, inhibit chondrocyte apoptosis in the knee joint, reduce the oxidative stress response and inhibit the degradation of the extracellular matrix of chondrocytes [33–36].

### Construction of the effective active ingredient pool and disease target set

The targets in the TCMSP database were searched, and a total of 513 targets for the RAB were included. The gene names of the targets were collected using the UniProt database, null and repetitive targets were deleted, and 101 effective active ingredient targets were obtained. A total of 2655 OA gene targets were obtained by searching and integrating the GeneCard, OMIM, PharmGkb, Therapeutic Targets database, and DrugBank database. A total of 101 compound targets were mapped to 2655 OA target genes to obtain 63 common target genes, as shown in Supplementary Table 3.

### Building a “ingredient-target network” network

**Fig. 2:**
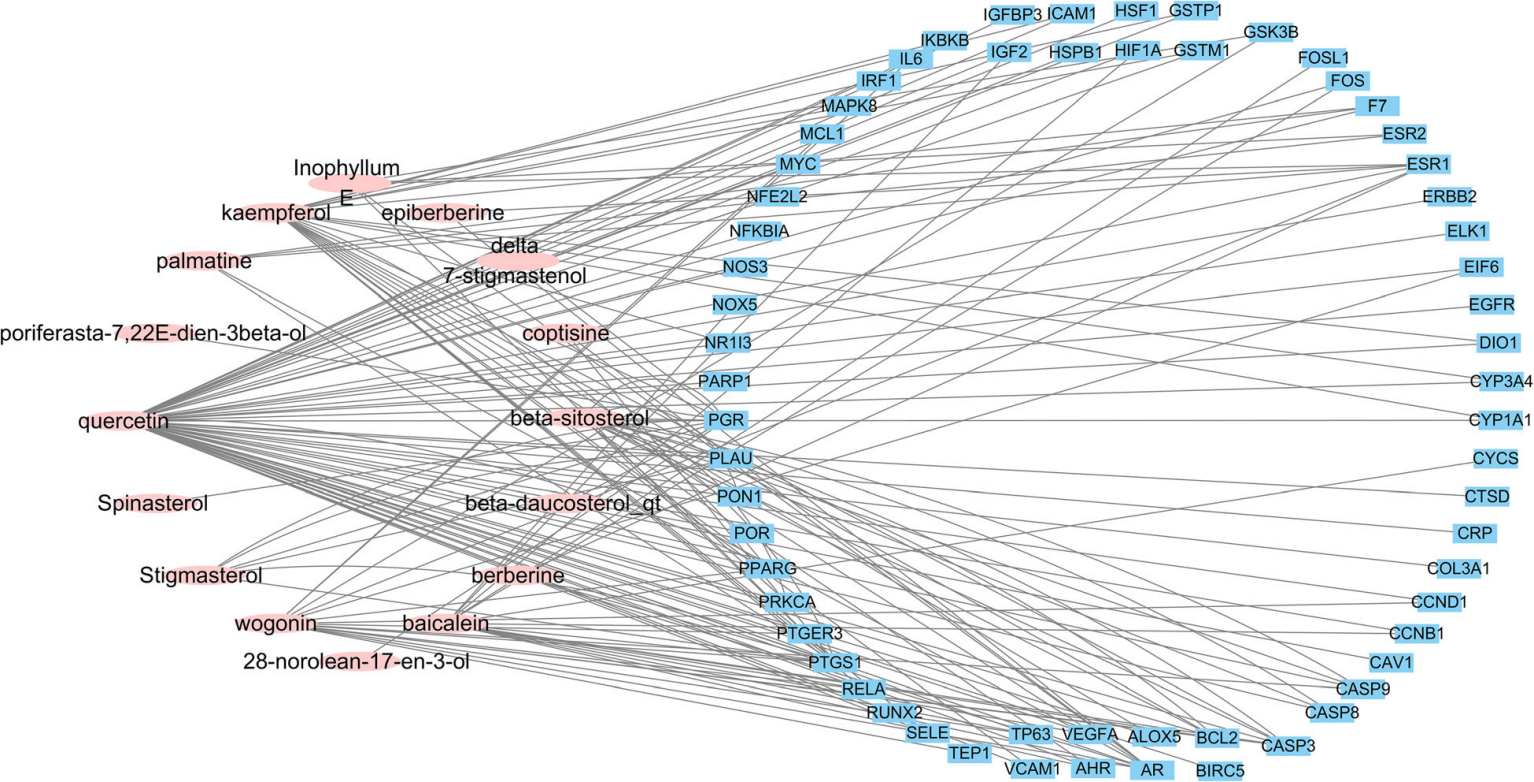
Ingredient-target network of Radix Achyranthis Bidentatae. The pink oval nodes are the main active ingredients of Radix Achyranthis Bidentatae, and the blue rectangle is the potential target for treating osteoarthritis of Radix Achyranthis Bidentatae

Sixteen drug components and sixty-three disease-related target screening results were mapped, chemical components without corresponding targets were removed, and duplicated targets were deleted. The data were imported into Cytoscape to construct an ingredient-target network, as shown in Fig. 2, and the docking results of RAB to the OA protein receptor were shown in Supplementary Table 4. (The octagonal node represents the active pharmaceutical ingredient, such as quercetin; the circular node represents the target of disease action, such as silk mitogen-activated protein kinase 8 (MAPK8).) As shown in Fig. 2, the network includes a total of 79 nodes and 151 sides. Different nodes represent the active constituents of RAB and the target of OA. Figure 2 shows that RAB can correspond to one or more active ingredients for one target, and multiple targets can correspond to the same active ingredient, suggesting that RAB has multi-component and multi-target characteristics for the treatment of OA.

### Target protein interaction network analysis

**Fig. 3:**
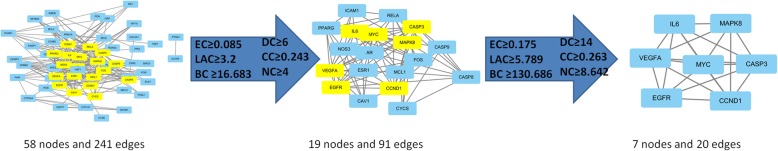
Process of topological screening for the PPI network

The common targets related to OA treatment of RAB were imported into the STRING database to obtain their interaction relationship. The interaction network has 58 nodes and 241 edges. TSV data were imported into Cytoscape, and CytoNCA was used to analyse the above PPIs based on DC, BC, CC, EC, NC, and LAC parameters. The thresholds for the first screening were Degree> 6, Eigenvector> 0.084871295, LAC> 3.2, Betweenness> 16.682932, Closeness> 0.243071465, and Network> 4, and the results showed a total of 19 nodes and 91 edges. Then, 19 targets were further screened. The second screening threshold was Degree> 14, Eigenvector> 0.17461096, LAC> 5.7894735, Betweenness> 130.68596, Closeness> 0.2626728, and Network> 8.641908. The second screening result was 7 nodes and 20 edges (Fig. 3), including MAPK8, IL6, VEGFA, EGFR, MYC, CCND1, and CASP3.

### GO and KEGG pathway enrichment analysis

**Fig. 4:**
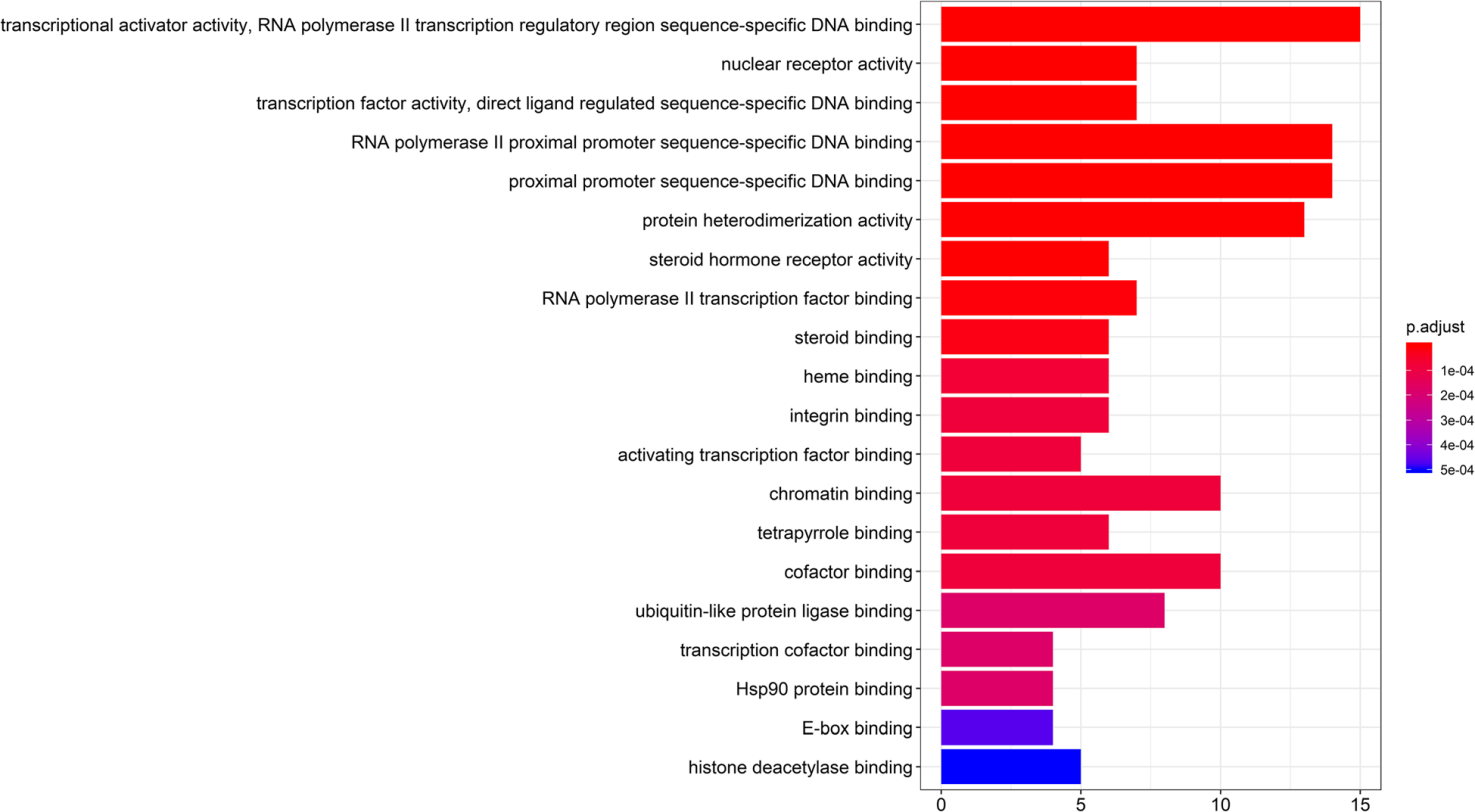
GO enrichment of Radix Achyranthis Bidentatae active components in the treatment of common targets of osteoarthritis

**Fig. 5:**
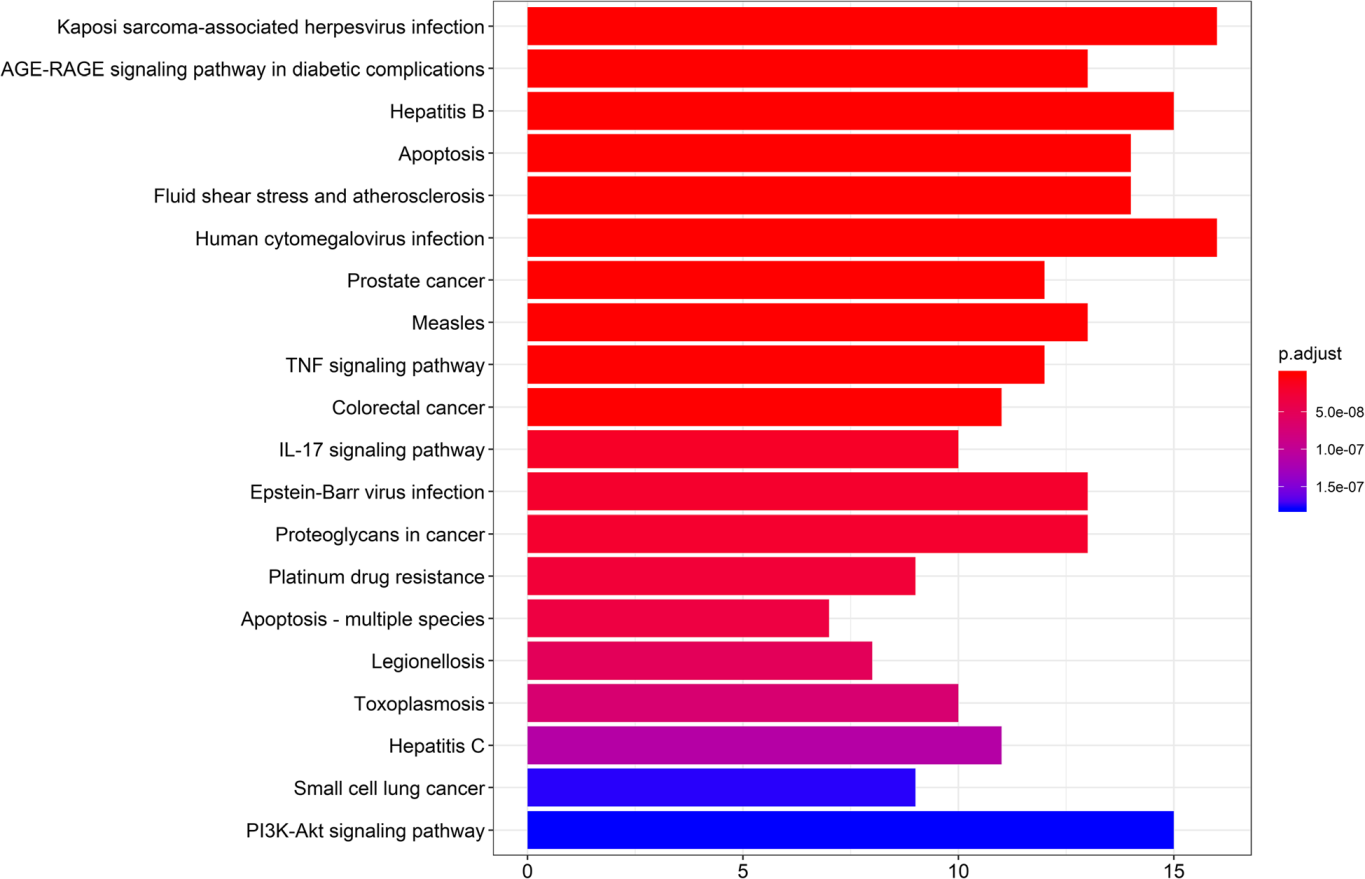
Enriched KEGG pathways of potential targets for treating osteoarthritis from the main active ingredients of Radix Achyranthis Bidentatae

The MF, BP and CC of 63 core targets were analysed, and the results showed that the key targets of RAB were highly enriched in 79 GO terms. The biological functions and processes involved, including specific sequence DNA binding, RNA polymerase II proximal promoter sequence-specific DNA binding, transcriptional activator binding, chromatin binding, cofactor binding, nuclear receptor activity, steroid hormone body activity, and nuclear chromatin binding, were closely related to biological processes, such as cell proliferation and apoptosis. The top 20 GO analysis results were screened, with *P* < 0.05 serving as the threshold, as shown in Fig. 4. KEGG enrichment analysis was performed on 63 targets of RAB. The results showed that the targets were enriched in 112 pathways, including viral-related signalling pathways, apoptosis signalling pathways, IL-17 signalling pathways, and PI3K/AKT signalling pathways. These classical signalling pathways play an important role in the occurrence and development of OA. The top 20 KEGG analysis results were screened, with P < 0.05 serving as the threshold, as shown in Fig. 5. GO and KEGG analyses suggested that RAB can act on OA through multiple pathways.

### Target path analysis

**Fig. 6:**
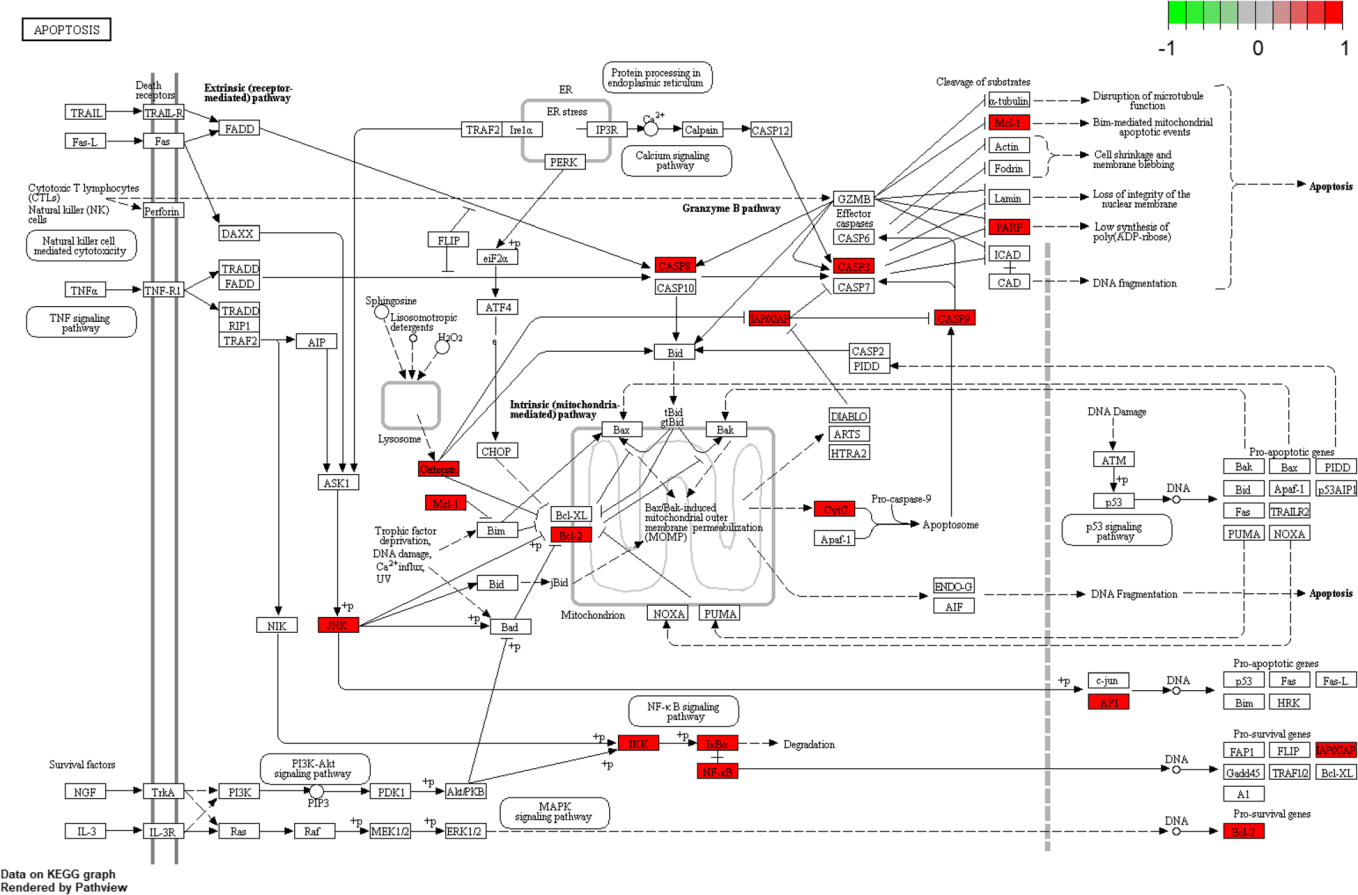
Pathway map of RAB in the treatment of osteoarthritis The main targets of Radix Achyranthis Bidentatae in the treatment of osteoarthritis are located in the apoptosis pathway. Arrows represent the activation effect, T-arrows represent the inhibition effect, and segments show the activation effect or inhibition effect.

The KEGG Mapper tool was used to obtain the pathway map of RAB for the treatment of OA, as shown in Fig. 6. The pathway targets were marked in white, and the targets of RAB for the treatment of OA were marked in red. The pathway map showed that RAB was involved in the treatment of OA, including the NF-κB signalling pathway and PI3K/AKT signalling pathway, with 17 effective targets of RAB in the treatment of OA. It was suggested that RAB may play a role in the treatment of OA by regulating several aspects, and its target may be located in these pathways.

### Molecular docking analysis

**Fig. 7:**
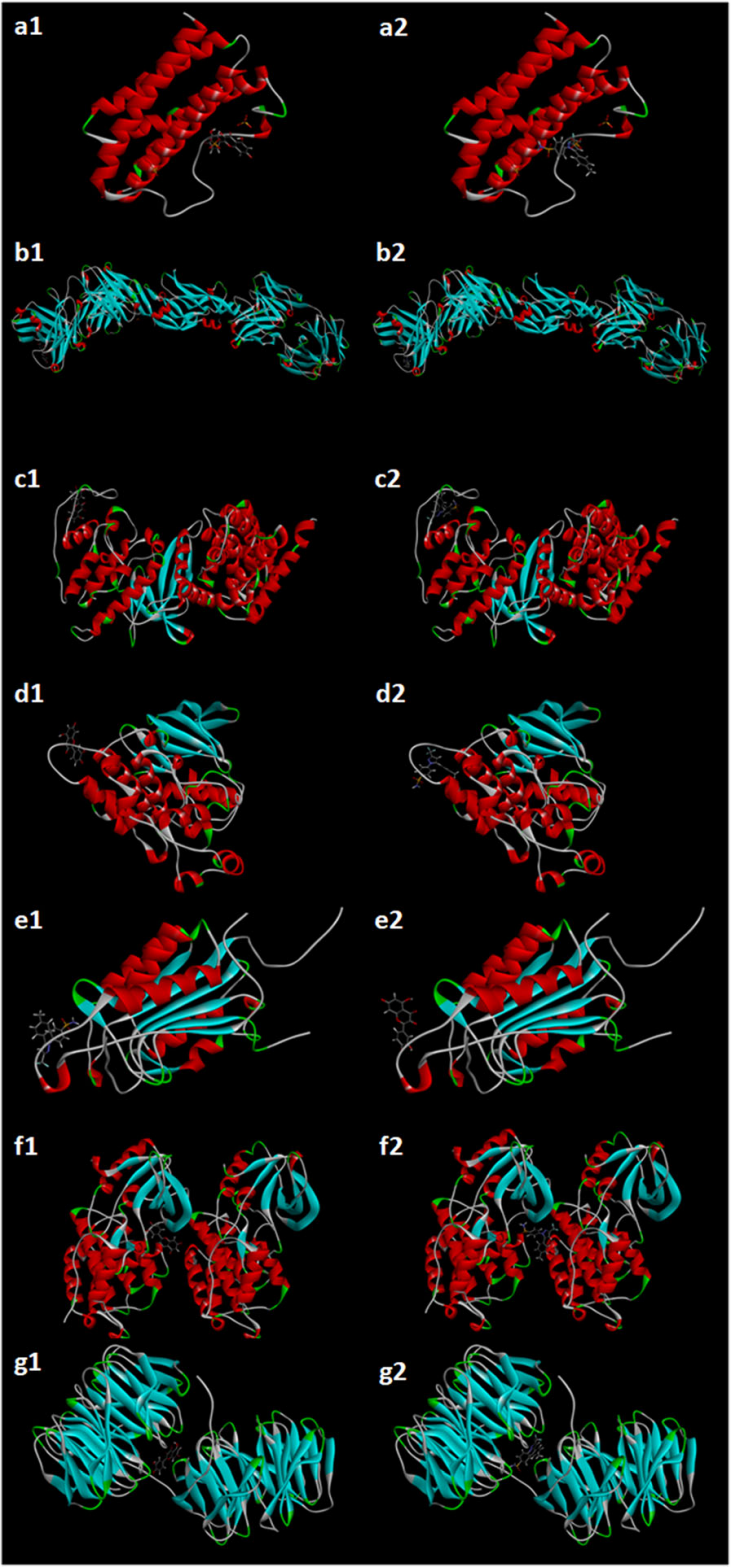
Molecular docking of compounds with core targets. (a1) Docking process of quercetin with IL6; (a2) Docking process of celecoxib with IL6; (b1) Docking process of quercetin with VEGFA; (b2) Docking process of celecoxib with VEGFA; (c1) Docking process of quercetin with CCND1; (c2) Docking process of celecoxib with CCND1; (d1) Docking process of quercetin with MAPK8; (d2) Docking process of celecoxib with MAPK8; (e1) Docking process of quercetin with CASP3; (e2) Docking process of celecoxib with CASP3; (f1) Docking process of quercetin with EGFR; (f2) Docking process of celecoxib with EGFR; (g1) Docking process of quercetin with MYC; (g2) Docking process of celecoxib with MYC

Discovery Studio software was used to validate network pharmacology by molecular docking. The LibDock score represents the degree of docking coincidence of molecules. The higher the score is, the better the binding of ligands to receptor proteins is. Molecular docking results showed that RAB had good affinity for the binding of active ingredients to key OA target protein molecules. Compared with the positive drug celecoxib, RAB had no significant difference in molecular docking fit. At the same time, these findings also indirectly verified that RAB had a regulatory effect on OA targets, such as CASP3 and MAPK8. The results indicated that the molecular docking results were consistent with the network pharmacology screening results, and the reliability of network pharmacology was verified by molecular docking. The docking results of RAB to the OA protein receptor were shown in Supplementary Table 5. The partial docking process was shown in Fig. 7.

## Discussion

RAB has anti-inflammatory, antitumour and immune-enhancing functions and has been widely used in the treatment of OA [37]. Studies have shown that RAB can delay the progression of OA and reduce the pathological damage of OA. The drug-containing joint fluid can improve cell viability, promote the proliferation of chondrocytes, increase the expression of type II collagen protein, and reduce the apoptotic rate of chondrocytes, thereby protecting cartilage [32–35]. Although the pharmacological effects of the main components of RAB have been studied in depth, the specific mechanism of their action in the treatment of OA has rarely been reported.

The degree of quercetin, kaempferol, wogonin, beta-sitosterol, baicalein and stigmasterol was the highest in the CHM-component-target-signalling pathway network. Quercetin inhibits ER stress by activating the sirtuin 1/adenosine monophosphate activated protein kinase (SIRT1/AMPK) signalling pathway, eliminates knee cartilage degeneration and reduces knee chondrocyte apoptosis. Quercetin also prevents inflammation and proteoglycan degradation by inhibiting the expression of TNF-α and MMP-9 [33]. Kaempferol has an anti-inflammatory effect and can reduce the production of inflammatory mediators by reducing the activation of p38 and ERK signalling pathways and inhibiting the NF-κB signalling pathway [38, 39]. Wogonin can inhibit the expression of matrix-degrading protease, enhance the expression of COL2A 1 and ACAN in cartilage, and disturb the balance between ROS and GSH levels. Wogonin also activates Nrf2 by inducing the ROS/ERK/Nrf2/HO-1-NQO1 signal axis [40]. Baicalein stimulated the expression of anti-apoptotic genes and reduced the production of pro-apoptotic and pro-inflammatory gene products in chondrocytes [40]. In addition, baicalein decreased the expression of protease in vitro and in vivo and the phosphorylation of p38 and ERK but did not decrease the phosphorylation of JNK [32]. Stigmasterol has anti-inflammatory and anti-catabolic properties [41]. Intra-articular injection of stigmasterol could decrease the expression of MMP-1, MMP-3 and MMP-13 in rabbit OA and upregulate the expression of TIMP-1 [31]. Reports on beta-sitosterol are limited. Therefore, quercetin, kaempferol, wogonin, baicalein and stigmasterol may be the most important components in the treatment of OA by RAB.

The top 8 targets of degree in the CHM-component-target-signal pathway network were MAPK8, IL-6, EGFR, VEGFA, CCND1, MYC, CASP3, and ESR1. The MAPK8 gene belongs to the MAPK signalling pathway and affects the proliferation and apoptosis of articular chondrocytes. By stimulating the MAPK signalling pathway, pro-inflammatory factors affect the level of MMPs and promote the development of OA [42]. IL-6 is a cytokine that strongly activates the immune system and enhances the inflammatory response. Recent studies have shown that IL-6 induces chondrocyte catabolism primarily through STAT3 signalling, and by blocking IL-6 or STAT3, it can alleviate the symptoms of OA mice caused by medial meniscus instability [43]. EGFR is highly expressed in healthy articular cartilage, promotes the secretion of lubricants in articular cartilage, and plays an important role in cell growth, proliferation and differentiation. The expression of the VEGFA gene was associated with early degenerative changes of cartilage and subchondral bone [44]. Studies have shown that synovial vascular endothelial growth factor may be involved in the pain pathway of KOA and may be mediated by apelin [45]. Overexpression of CCND1 reversed the effect of FOXD2-AS1 inhibition on chondrocyte viability [46]. Related studies have shown that Cyclin D1 (a protein encoded by the human CCND1 gene) silencing inhibits IL-1β-induced proliferation of rat chondrocytes induced by OA and induces apoptosis [47]. The MYC gene is a group of previously characterized oncogenes, including C-myc, N-myc, and L-myc. A study found that C-myc gene silencing promotes cell proliferation and inhibits IL-1β-induced rat chondrocyte apoptosis and cytokine expression [48]. CASP3 was determined to play an important role in the apoptosis of articular chondrocytes in KOA rats. The expression of CASP3 is positively correlated with the apoptosis of articular chondrocytes [49]. The ESR1 gene can be expressed in osteoblasts, osteoclasts, chondrocytes and bone marrow stromal cells, as well as bone cells. E2 targeting activation of ESR1 in chondrocytes can inhibit ERK phosphorylation activation, thereby inhibiting the ERK signalling pathway, promoting the formation of autophagic flow, activating autophagy, reducing apoptosis, and causing chondrocyte proliferation [50].

The results of enrichment analysis showed that the main pathways involved in the treatment process are cell apoptosis, the PI3K/AKT pathway, the IL-17 signalling pathway, and viral infection. Apoptosis is the programmed death of cells. Modern studies have found that the signalling pathways that regulate the apoptosis of OA chondrocytes are complex and diverse. Qin et al found that specific inhibition of the SDF-1/CXCR4 signalling pathway can regulate subchondral bone microstructure and attenuate OA pathological damage [51]. Kalaitzoglouë observed that the activation of the TLR4 signalling pathway induces cartilage catabolism in middle-aged female mice, thereby aggravating the progression of OA [52]. MAPK is a serine/threonine protein kinase in cells. As one of the important signal transduction pathways in eukaryotic cells, MAPK participates in the proliferation, differentiation and apoptosis of chondrocytes, induces the secretion of matrix metalloproteinases and causes cartilage matrix degradation. The abnormal expression of MAPK can accelerate the process of OA [53].

The PI3K/AKT pathway is an important anti-apoptotic signalling pathway in vivo that can promote the apoptosis and autophagy of chondrocytes and play an important role in the development of OA [54, 55]. AKT, a serine/threonine protein kinase, is activated by PI3K and recruited to the plasma membrane, which plays an important role in regulating cell growth and apoptosis. Recent studies have shown that AKT activation can regulate autophagy and improve cartilage injury and related indicators.

IL-17 plays an important role in OA. Accurate and effective regulation of IL-17 signal transduction can prevent inflammation. A large number of studies have shown that IL-17 is overexpressed in the synovium of RA patients, but there are few studies on the expression of IL-17 in OA patients and its relationship with the severity of arthritis [56–58]. Some scholars have found that the level of IL-17 is related to the severity of knee pain in osteoarthritis. Blocking the IL-17 signalling pathway can delay the pain associated with osteoarthritis [59]. A meta-analysis also showed that the pathogenesis of KOA in the Chinese Han population may be positively correlated with IL-17A (rs2275913) [60].

This study has a number of limitations. There are notably few studies investigating the relationship between viral pathways and OA. These results may be observed because the current research data on virus-related pathways are more abundant, and the analysis in the existing database will produce certain bias. Therefore, virus-related pathways, such as Kaposi’s sarcoma-associated herpesvirus infection, hepatitis B, hepatitis C, human cytomegalovirus infection and other viral-related pathways, can also be involved in the treatment of OA.

In summary, this study used network pharmacological methods and techniques to identify 17 kinds of drug components and 101 potential targets in RAB. There were 2515 OA disease-related targets, 63 of which were RAB. Through enrichment analysis of GO biological processes and KEGG signalling pathways, it was preliminarily predicted that RAB may regulate the targets of MAPK8, IL-6, VEGFA, CCND1, CASP3, and ESR1 through quercetin, kaempferol, wogonin, beta-sitosterol, baicalein, and stigmasterol. The regulation of apoptosis signalling pathways, IL-17 signalling pathways, and PI3K/AKT signalling pathways ultimately inhibit the inflammatory response, regulate immune function and regulate cell apoptosis to treat OA. Due to the limitations of database data and corresponding analysis algorithms and software functions of various platforms, confirming these results requires experimental research, and the specific mechanism governing these phenomena also needs to be elucidated by experimental research.

## Conclusions

This study investigated the effective active ingredients and molecular mechanisms of RAB in the treatment of OA from the perspective of network pharmacology. The active ingredients of RAB in OA treatment are composed of 16 compounds; among them, quercetin, kaempferol, wogonin, beta-sitosterol, baicalein and stigmasterol are the important active ingredients. There are 63 target genes involved in the treatment of OA by RAB, among which MAPK8, IL-6, EGFR, VEGFA, CCND1, MYC, CASP3 and ESR1 are the key target genes. The signalling pathways of RAB in the treatment of OA mainly include the apoptosis signalling pathway, the IL-17 signalling pathway and the PI3K/AKT signalling pathway. In addition, the results of this study provide a new way to further study the mechanism of RAB in the treatment of OA.

## Supplementary information

**Additional file 1.** Supplementary Table 1: Potential target information of RAB for OA.

**Additional file 2.** Supplementary Table 2: Active ingredient parameters of RAB.

**Additional file 3.** Supplementary Table 3: Common gene of RAB in the treatment of OA.

**Additional file 4.** Supplementary Table 4: Compound-target pair information.

**Additional file 5.** Supplementary Table 5: Results of ligand-receptor protein molecular docking.

## Data Availability

The datasets used and/or analysed during the current study are available from the corresponding author on reasonable request.

## Abbreviations

RAB: Radix achyranthis bidentatae
CHM: Chinese herbal medicine
OA: Osteoarthritis
TCMSP: traditional Chinese medicine systems pharmacology
OB: Oral bioavailability
DL: Drug-likeness
KEGG: Kyoto encyclopedia of genes and gnomes
PDB ID: Protein data bank identification
PPI network: Protein-protein interaction network
MF: Molecular function
BP: Biological process
CC: Cellular component
