# Supplementary material for: Network Pharmacology Approach to Uncover the Mechanism Governing the Effect of Radix Achyranthis Bidentatae on Osteoarthritis
Source: BMC Complement Med Ther. 2020 Apr 21;20:121. doi: 10.1186/s12906-020-02909-4 (PMC7171799; doi:10.1186/s12906-020-02909-4)
Supplement: Supplementary file 2 — Additional file 2. Supplementary Table 2: Active ingredient parameters of RAB. [file 12906_2020_2909_MOESM2_ESM.docx]

| **MOL ID** | **Structure** | **Name** | **OB** | **DL** |
| --- | --- | --- | --- | --- |
| MOL001006 | 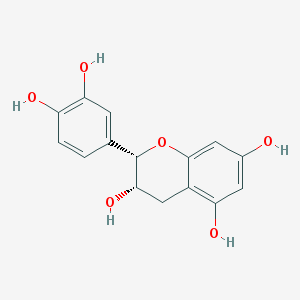 | poriferasta-7,22E-dien-3beta-ol | 42.98 | 0.76 |
| MOL000098 | 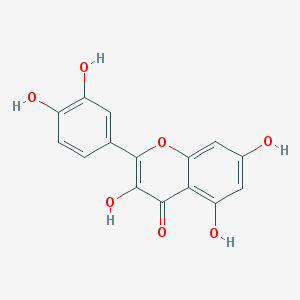 | quercetin | 46.43 | 0.28 |
| MOL000358 | 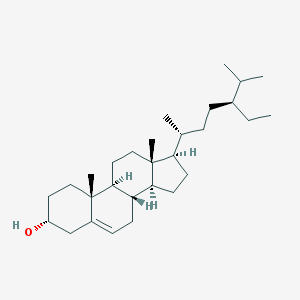 | beta-sitosterol | 36.91 | 0.75 |
| MOL012461 | 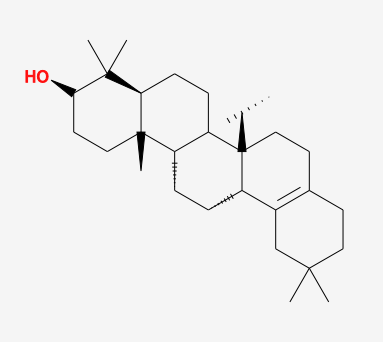 | 28-norolean-17-en-3-ol | 35.93 | 0.78 |
| MOL000422 | 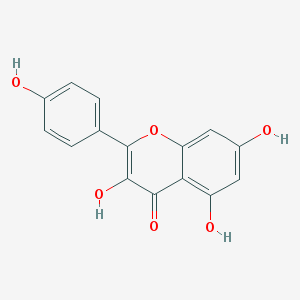 | kaempferol | 41.88 | 0.24 |
| MOL000449 | 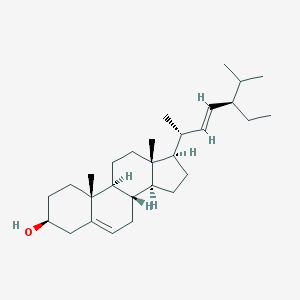 | Stigmasterol | 43.83 | 0.76 |
| MOL000173 | 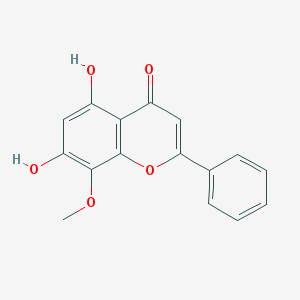 | wogonin | 30.68 | 0.23 |
| MOL000785 | 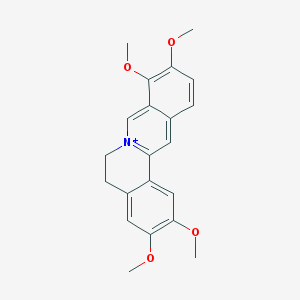 | palmatine | 64.6 | 0.65 |
| MOL002714 | 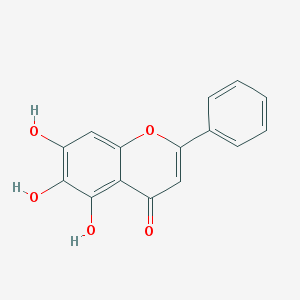 | baicalein | 33.52 | 0.21 |
|  |  |  |  |  |
| MOL003847 | 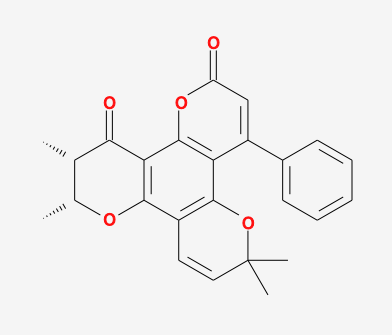 | inophyllum E | 38.81 | 0.85 |
| MOL004355 | 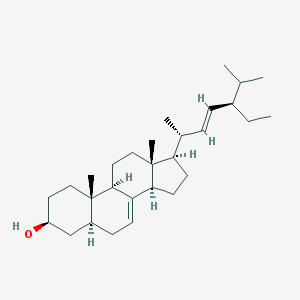 | spinasterol | 42.98 | 0.76 |
| MOL001454 | 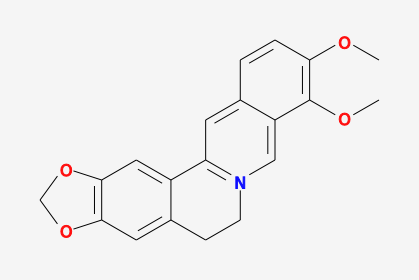 | berberine | 36.86 | 0.78 |
| MOL001458 | 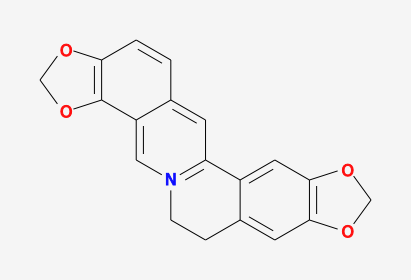 | coptisine | 30.67 | 0.86 |
| MOL002643 | 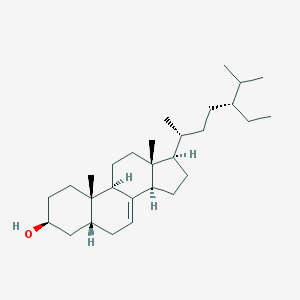 | delta 7-stigmastenol | 37.42 | 0.75 |
| MOL002897 | 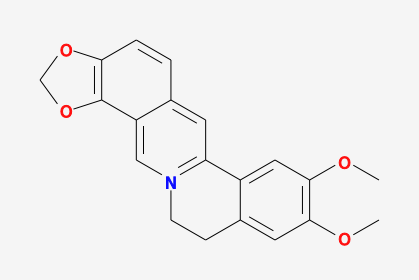 | epiberberine | 43.09 | 0.78 |
| MOL000085 | 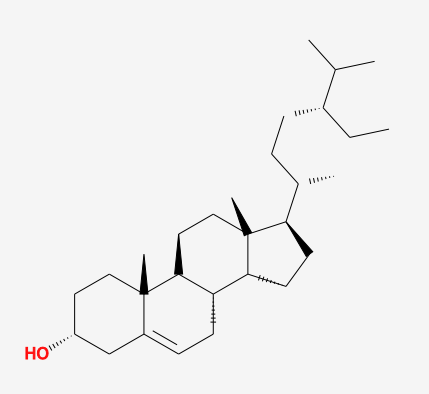 | beta-daucosterol_qt | 36.91 | 0.75 |

**Supplementary Table 2:** **Active ingredients parameters of RAB.**

* Oral Bioavailability: OB; Drug-Likeness: DL
